# Standardization of bone morphometry and mineral density assessments in zebrafish and other small laboratory fishes using X-ray radiography and micro-computed tomography

**DOI:** 10.1093/jbmr/zjae171

**Published:** 2024-10-30

**Authors:** Erika Kague, Ronald Young Kwon, Björn Busse, Paul Eckhard Witten, David Karasik

**Affiliations:** Institute of Genetics and Cancer, Centre for Genomic and Experimental Medicine, University of Edinburgh, Edinburgh EH4 2XU, United Kingdom; Department of Orthopedics and Sports Medicine, University of Washington School of Medicine, Seattle, WA 98105, United States; Institute for Stem Cell and Regenerative Medicine, University of Washington, Seattle, WA 98109, United States; Department of Osteology and Biomechanics, University Medical Center Hamburg-Eppendorf, 22529 Hamburg, Germany; Interdisciplinary Competence Center for Interface Research (ICCIR), University Medical Center Hamburg-Eppendorf, 22529 Hamburg, Germany; Evolutionary Developmental Biology, Department of Biology, Ghent University, Ghent 9000, Belgium; The Musculoskeletal Genetics Laboratory, The Azrieli Faculty of Medicine, Bar-Ilan University, Safed 1311502, Israel

**Keywords:** zebrafish, X-ray radiography, micro-computed tomography, skeletal nomenclature, skeletal reporting

## Abstract

Zebrafish and other small laboratory fishes are emerging as important animal models for investigating human skeletal development and diseases. In recent years, there has been a notable increase in research publications employing X-ray radiography and micro-computed tomography to analyze the skeletal structures of these animals. However, evaluating bone morphology and mineral density in small laboratory fish poses unique challenges compared to well-established small rodent models. The varied approaches to image acquisition, analysis, and reporting across studies have led to substantial obstacles in interpreting and comparing research findings. This article addresses the urgent need for standardized reporting of parameters and methodologies related to image acquisition and analysis, as well as the adoption of harmonized nomenclature. Furthermore, it offers guidance on anatomical terminology, units of measurement, and the establishment of minimal parameters for reporting, along with comprehensive documentation of methods and algorithms used for acquisition and analysis. We anticipate that adherence to these guidelines will enhance the consistency, reproducibility, and interpretability of reported measurements of bone density and morphometry in small fish models. These advancements are vital for accurately interpreting phenotypes and gene functions, particularly in the context of multi-center studies.

## Purpose

Small teleosts, such as zebrafish (*Danio rerio*) or medaka (*Oryzias latipes*) are widely used in biomedical research as models for human skeletal diseases, as recently reviewed.^1^ Zebrafish skeletal cells are homologous with human skeletal cells, exhibiting osteoblasts, osteocytes, osteoclasts, and chondrocytes.^2^ Advantages, including their transparency during early development, external fertilization, fecundity, low cost, small size, and ease of genetic manipulation, make them valuable animal models.^3^ Moreover, over 70% of human genes have at least one zebrafish ortholog, therefore can be studied in zebrafish.^4^ We expect increased use of teleosts in biomedical research, to validate findings from human genomic studies, such as genome-wide association and whole genome sequencing studies.^3^^,^^5^

High-resolution 2D X-ray imaging, 3D X-ray microscopy, and 3D micro-computed tomography (μCT), allowing to evaluate bone mass, shape, and mineral density, are increasingly used for researching human skeletal diseases in zebrafish and medaka.^6–9^ Standard guidelines for assessing bone microstructure in rodents using μCT have been established.^10^^,^^11^ They are, however, not directly suitable for small fish due to anatomical and microstructural differences.^2^ This leads to inconsistent reporting in fish studies, including differences in terminology, methods for data acquisition, and reported outcomes.

To promote uniformity and reproducibility in the analyses of small fish models in skeletal research, we established a committee with experts from Europe and the United States. Our diverse expertise covers skeletal imaging, genetics, and evolutionary biology. Our main goal is to propose initial guidelines for consistent reporting of methods and parameters related to nomenclature, X-ray-based imaging, and analysis, aligning with the latest *ARRIVE (Animal Research: Reporting of In Vivo Experiments) guidelines* for reporting animal experiments.^12^ While our focus is on zebrafish and medaka, our guidelines will benefit skeletal studies in other small teleosts, like the killifish *Nothobranchius furzeri*, a model for skeletal aging.^2^ Our proposed guidelines stand out as an initiative open for future discussions before an international consensus on the gold standard is reached.

## Overview of 2D X-ray and 3D μCT imaging and their use for skeletal phenotyping in small fish

### Use of X-ray radiography for skeletal phenotyping in small fish

X-ray radiography has long been utilized for imaging bones in small teleost fishes.^13^ In 2002, zebrafish skeletons were imaged using mammographic systems like the GE Senograph 600T.^14^ Nowadays, digital cabinet X-ray systems offer comparable resolution to film with the added benefits of immediate image viewing and unlimited exposures, facilitating real-time imaging.^14^ However, minimizing radiation, a requirement in human and veterinary medicine, can reduce system resolution.

A 2D X-ray has also been used to infer bone density. Ell and Sprigg^15^ conducted research to determine the radiodensity of fish bones from multiple species. Variation in radiodensity using X-rays was then demonstrated in *sp*7^-/-^ and in *ctsk* somatic mutants,^8^ and under restriction of dietary phosphorus.^16^ The rapid image acquisition in 2D X-ray allows for non-invasive longitudinal studies and rapid-throughput screening, which can be challenging with μCT due to longer acquisition times. For instance, Fisher *et al.*^17^ used X-rays for whole-skeleton phenotypic screening of mutagenized fish, leading to the identification of the Chihuahua (*col1a1a^-/-^*) mutant, the first zebrafish model for osteogenesis imperfecta.

**Figure 1:**
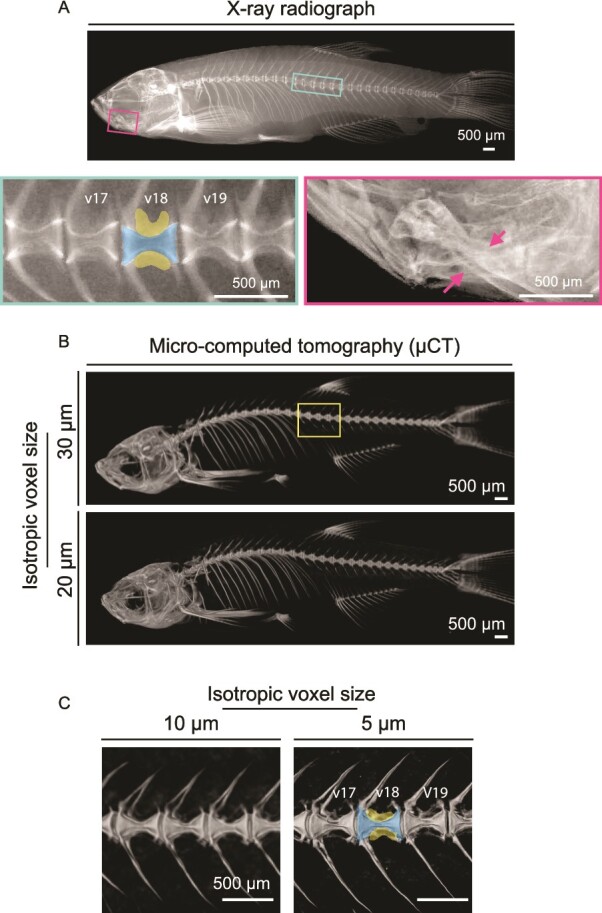
Examples of images acquired using 2D X-ray and 3D μCT. (A) 2D X-ray image of adult fish *ex vivo.* Regions within boxes were zoomed in (v17 v18 and v19 are indicated). The centrum region of the v18 is highlighted in blue and the trabecular-like bone plate region in yellow. Magenta arrows point to overlapping bones in the image, demonstrating a limitation of X-ray images. (B) μCT of whole-body adult fish (3-mo-old) scanned at different isotropic voxel size (30 and 20 μm). (C) The region highlighted within the square in B was scanned with isotropic voxel size of 10 and 5 μm. Note that trabecular bone-like plates are better visualized at 5 μm. The centrum region of the v18 is highlighted in blue and the trabecular-like bone plate region in yellow. Scale bars are shown in each picture. μCT images were rendered using Amira (FEI).

A typical commercial digital 2D X-ray system, designed for small rodent imaging, may report a nominal resolution of 6 μm at 4X geometric magnification, and a focal spot size of 11 μm, with energy ranging from 10 to 50 kV (1 mA maximum). Such resolution allows for clear viewing of, for example, vertebral centra, arches, and some smaller skeletal features in adult fish (Figure 1A). However, it may not fully resolve small intramuscular bones or bone structures in juvenile stages. Additionally, due to the 2D nature of the images, overlapping bones cannot be distinguished, and trabecular-like bone plates may not be detected (Figure 1A).

### Use of μCT for skeletal phenotyping in small fish

μCT has emerged as a powerful approach to evaluate the 3D microarchitecture of the fish skeleton, with precise and reproducible bone density measurements (Figure 1B). Desktop μCT scanners achieve an isotropic voxel size as small as 3 μm. Systems optimized for small rodents generally have sufficient resolution to detect compact bone, but not all small bone structures can be detected. In some cases, output from small skeletal structures in adult zebrafish is numerically comparable to adult mouse. For instance, Hur *et al.*^6^ found similar values of thickness of the centrum, haemal arch, and neural arch (50-80 μm) and tissue mineral density (TMD) (400-600 mg HA/cm^3^) with that of mouse trabeculae (20-70 μm and 600-700 mg HA/cm^3^).^18^

Reported outcomes for μCT analysis and the methods used for their assessment are continuously evolving in fish studies. While μCT was introduced to zebrafish in 2010,^19^ it was only from 2017 that its use significantly increased. Researchers like Hur *et al.*^6^ developed comprehensive measurement techniques using semi-automated software, enabling analysis of multiple vertebrae elements and TMD, resulting in numerous measurements per animal. Others, such as Charles *et al.*,^9^ focused on parameters like bone volume to tissue volume ratio and BMD of the centra. Kague *et al.*^8^ utilized computational analysis to automate vertebral segmentation, facilitating volumetric measurements and TMD analysis on large datasets. However, discrepancies in outcomes, nomenclature, and reporting parameters (Supplementary Table SS1) remain a notable issue in the literature, deviating from ARRIVE guidelines.^12^ Therefore, adopting consistent nomenclature and reporting standards is crucial for enhancing reproducibility and comparability in fish skeletal studies, as outlined below.

## Recommendations for image acquisition

We describe key considerations and suggest standardization for 2D X-ray (when applicable) and 3D μCT image acquisition. Furthermore, we provide guidance regarding essential details that should be incorporated into the Methods section of a research publication (Tables 1 and 2).

**Table 1:** Recommended parameters for reporting 2D X-ray and 3D μCT image acquisition.

Category	2D X-ray parameter	3D μCT parameter
**Sample preparation**	Method used for sample preparation (eg, freshly euthanized, frozen, or fixed)	Method used for sample preparation (eg, freshly euthanized, frozen, or fixed)
**Sample mounting and positioning**	Whether the samples were imaged *in vivo* or *ex vivo* in whole animals or dissected tissues Imaging medium	Whether the samples were imaged *in vivo* or *ex vivo* in whole animals or dissected tissues Scanning medium Whether multiplexed scanning was performed, and if so, how many animals were imaged simultaneously
**X-ray source**	X-ray tube potential (kV)	X-ray tube potential (kV)
	X-ray tube current (μA)	X-ray tube current (μA)
	Whether filtering was performed to reduce beam hardening and if so the type of filter used	Whether filtering was performed to reduce beam hardening and if so, type of filter used
**X-ray detector**	Exposure time (s) Distance between the X-ray source and the specimen (cm) Number of frames used for averaging Pixel size (μm) Detector size (mm) and mega pixel number	Integration time (ms) Number of frames used for averaging Number of frames per projection Voxel size (μm)
**Phantom calibration**	Calibration method used for estimating bone density (eg, aluminum wedge)	Calibration method used for measuring bone density
**Other**	Machine make and model	Machine make and model

Abbrevations: μCT, micro-computed tomography.

**Table 2:** Recommended parameters for reporting μCT image segmentation, thresholding, image analysis and others.

**μCT image segmentation and thresholding**	
Category	Parameter
**Bone density**	Whether BMD and/or TMD was examined.
	If BMD is reported, a description of the volume of interest used for its calculation should be provided with enough detail for readers to replicate the analysis.
**Morphometry (landmark-based)**	A description of how the landmarking was performed should be provided with sufficient detail for readers to replicate the analyses. When conducting manual landmarking, such details should include: A list or schematic detailing anatomical features where landmarks were placed. A description of how quantities were calculated. A description of how landmarks were placed.
**Morphometry (segmentation-based)**	For segmentation-based methods, in addition to details on segmentation and thresholding, a description of the methods used for quantification of measures should be provided. Details should include the functions and software used for calculation If bone volume (BV)/tissue volume (TV) is reported, a description of the volume of interest used for its calculation should be provided with enough detail for readers to replicate the analysis.
**μCT image analysis**	
**Segmentation**	Whether segmentation was performed by defining an ROI and thresholding, or contouring bones in unthresholded images. Details about whether the Region of Interest (ROI) was generated through the manual delineation of contours or by defining a geometric shape (eg, a box or cylinder), including any reference to landmarks employed in ROI specification. If ROIs were automatically generated, methodological details on how they were specified.
**Thresholding**	The global threshold (mg HA/cm^3^) used for segmentation, if manually selected. The algorithm used for threshold calculation if the threshold was calculated automatically.
**Other recommended parameters for reporting**	
**Age and standard length**	Report the standard length of the animals used for the study (eg, ~30 mm SL) along with age.
**Strain and sex**	Background strain: describe what zebrafish strain was used in the experiment. When analyzing mutants, describe the background strain. Report whether studies were performed in male, female, or mixed-sex groups.
**Experimental controls**	Controls (eg, clutch mate controls) used for experimental groups.
**Normalization**	If normalization is performed, a description of the normalization method and justification for the chosen approach.
**Images**	Type of image (eg, max intensity projection, or surface rendering from segmented data) should be reported in figure caption. Details on how the image was generated. Oriented to keep with accepted convention within zebrafish research (eg, in the lateral view, anterior end on the left and dorsal side up).

Abbrevations: μCT, micro-computed tomography; SL, standard length; TMD, tissue mineral density.

### Sample preparation

Freshly euthanized, frozen, or fixed specimens can be used depending on desired outcomes and subsequent processing. Fresh or frozen tissues are advantageous for biomechanical testing; however, they do not preserve the histology. Samples should be frozen by placing them at −80 °C, and they should be thawed before scanning to avoid motion artifact. We recommend using PFA fixation, which works well with post-μCT histological processing. An incision in the abdomen before fixation will ensure thorough PFA penetration. Fish should be incubated in 4% PFA for 24 h under gentle agitation (4 °C), with the PFA volume at least five times the total fish volume. After fixation, wash samples in tap water for 1 h to remove fixative, then in serial ethanol solutions (25%, 50%, and 70%) for 1 h each. Although ethanol may cause small shrinkage, mainly soft tissue, samples can be maintained in 70% ethanol at 4 °C. Consistency in sample preparation across groups is crucial for comparisons, as different fixations can result in variation of readout measurements. Sample preparation should be detailed in the Methods section.

### Sample mounting and positioning

2D X-rays can be performed *in vivo* or *ex vivo* (freshly euthanized, frozen, or after fixation)*.* For *in vivo* imaging, the fish may be lightly anesthetized with Tricaine methanesulfonate (MS222) solution (0.168 mg/L in fish system water).^20^ Samples should be positioned straight and as flat as possible. Multiple samples can be scanned simultaneously, allowing a gap of about 1 cm between fish. Whether the samples were imaged *in vivo* or *ex vivo* in whole animals or dissected tissues should be reported in the Methods.

3D μCT scanning of zebrafish is predominantly performed *ex vivo* because of longer acquisition times and challenges with long-term anesthesia in zebrafish. Adult zebrafish in Tricaine solution typically experience respiratory arrest in 20 min or less,^21^ often shorter than scan acquisition times. Research into long-term anesthesia methods for zebrafish is ongoing.^22^

For μCT, specimens should be positioned with the anteroposterior axis parallel to the axis of rotation of the scanner. For mounting, one method is to roll zebrafish in strips of wet paper towel, which can be supplemented with a surrounding wrap of parafilm. This stabilizes zebrafish within the specimen holder in a mostly air-filled space and helps to keep the sample hydrated during long scans. This can also help to minimize motion artifacts, which is particularly important when performing high-resolution scanning. The sample can also be scanned in air by wrapping animals in a strip of dry paper towel. A small piece of porous plastic tape, such as 3 M transpore tape, can be used to keep the wrapped paper towel from unfurling.

To increase throughput, fish can be subjected to a “multiplexed scanning,” albeit at the expense of resolution.^6^ In this approach, multiple fish (usually 2-4 and occasionally up to 8) are arranged in an array format to allow simultaneous scanning.^6^ Including both controls and mutants in the same bulk scan can also help reduce the batch effect in density between groups. Wrapping the fish samples with paper towels also facilitates automated segmentation of individual animals in bulk scans obtained by multiplexed scanning.^6^ Whether multiplexed scanning was performed, and if so, how many animals were scanned simultaneously, should be reported.

Animals can be scanned in various media, including air or liquid (eg, saline, ethanol, or neutral-buffered formalin). Because X-ray attenuation through liquid media is similar to lean tissue, scanning animals in air is advantageous for situations in which automatic segmentation of soft tissue from the surrounding medium is necessary, such as lean mass quantification, as well as segmentation of individual fish from multiplexed (bulk) μCT scans.^6^ Importantly, the scanning medium can affect the relationship between attenuation coefficients and TMD.^23^ For bone, the attenuation properties of the medium are not as critical as for soft tissues, where scanning in an aqueous medium can lead to loss of contrast. Thus, it is recommended that the same scanning medium be used across an entire experiment. The scanning medium should be reported in methods.

### X-ray source

Tube voltage governs the energies of the X-ray photons that are generated and thus influences image contrast. Lower energies increase contrast and may be useful when analyzing younger fish whose skeletons are not well mineralized. In contrast, higher energies may be useful for thick or dense samples in which there is excessive X-ray attenuation. Both 2D X-ray and μCT studies should report the X-ray tube potential, preferably in units of kilovolts (kV) or kilovolts peak voltage (kVp). Prior 2D X-ray studies reported from 17-20 kV^17^ to 45 kV.^8^^,^^24^ Prior μCT studies of adult zebrafish bones have reported X-ray tube potentials from 45 to 130 kV.^6^^,^^8^^,^^25–28^ Young and aged fish can be scanned with the same parameters, starting from 3-mo-old.

X-ray tube current governs the number of the X-ray photons generated per unit time. Increasing the tube current is expected to increase the signal-to-noise ratio since more X-ray photons have the potential to be averaged over for a given projection. Studies should report the tube current, preferably in units of microamperes (μA). Prior 2D X-ray studies of adult zebrafish reported 20^14^-46 mA.^8^^,^^24^ μCT studies have reported X-ray tube currents of 80-200 μA.^9^^,^^29^

### Beam hardening and X-ray filters

Beam hardening is the phenomenon by which the average energy of the X-ray beam is increased due to the filtering of lower-energy X-ray photons, which are more easily attenuated than higher-energy X-ray photons.^11^ A common image artifact arising from beam hardening is termed “cupping”^30^; for a uniform cylindrical sample, cupping manifests as higher attenuation in the middle of the cylinder compared to the edges. To mitigate beam-hardening effects, the beam can be pre-hardened by placing a filter in the X-ray path, like a thin aluminum or copper filter. Further filtering can be performed on the sample by immersing it in a liquid scanning medium. Finally, beam hardening effects can be reduced during reconstruction. Studies should report methods used to account for beam hardening. μCT studies should report whether filtering was used and what type.

### X-ray detector

One key control parameter for the X-ray detector is the signal-to-noise ratio, influenced by the rate of X-ray photons collected per unit time. This number is influenced by integration time and frame averaging, which involves repeating and averaging across frames. Scan duration is affected by these variables, along with the number of projections per revolution, and can be optimized for a balance between scan time and image quality. It is important to report integration time, frame averaging details, and preferably in milliseconds (ms). Prior μCT studies of adult zebrafish bones have reported integration times of 114-1500 ms.^9^^,^^19^ 2D X-ray studies should also report the exposure time, preferentially in seconds (s). Prior 2D X-ray studies reported from 3-4^17^ to 10 s.^16^

### Voxel size and resolution

A voxel is a 3D pixel in the rendered μCT image. For cubic voxels, the length of one of the sides of the voxel is called (isotropic) voxel size. Partial volume effect refers to the phenomenon in which a voxel possesses more than one type of tissue, resulting in an attenuation value that reflects the proportional contribution of each of the underlying tissues within the voxel. Ideally, the smallest achievable voxel size would be used in all cases to minimize the influence of partial volume effects. However, smaller voxel sizes generally come at the expense of smaller fields of view and longer scan durations. In general, the ability to resolve small details of the fish skeleton, such as vertebral “trabecular-like” bone plates, will become more difficult as voxel size is increased (Figure 1B and C).

Regarding the ratio of the object’s size being analyzed to the voxel size, the minimum recommended ratio is two.^11^ Local errors of tissue identification/borders may be substantial when the ratio is close to two, though they will be reduced when averaged across the entire structure.^11^ Voxel sizes of 6-21 μm to analyze morphological features of the adult zebrafish spine have been reported^6^^,^^9^^,^^25^ and 2.6 μm to generate μCT-based finite element models of individual vertebrae.^29^ Hur *et al.*^6^ found that, relative to scanning at 10.5 μm voxel size, scanning at 21 μm voxel size systematically reduced TMD and increased thickness in the centrum, haemal arch, and neural arches, which had an averaged thickness of 50-80 μm.^6^ However, these authors found an extremely high correlation (*R*^2^ > 0.99) in TMD and thickness measures scanned at 10.5 and 21 μm.^6^ Choosing the appropriate voxel size requires careful consideration of the size of the object being analyzed, as well as the experimental objectives.

It is necessary to understand the relationship between voxel size and resolution to interpret μCT images. Resolution refers to the smallest distance between objects in which they can still be identified to be independent and is typically reported as a characteristic size (μm) or a spatial frequency (assessed by resolving lines on a grid, and thus reported as line pairs per mm).^11^ Nominal resolution is the smallest possible voxel size of a scanner’s reconstructed image and thus can be equated to the voxel size. The resolution of the μCT system cannot be greater than the voxel size/nominal resolution and is almost always less. This is for a variety of reasons, including image artifacts generated during acquisition,^30^ physical sources of blur, such as motion, dispersion of light within the scintillator and detector, or image filtering processes, during reconstruction. Methods for determining resolution include those based on measuring the modulation transfer function (a parameter derived from imaging a small point or thin line and which is often used to characterize the resolution of optical systems) of the instrument, or imaging objects with a known geometry and determining the smallest recognizable object by eye. In reporting spatial resolution, a standardized approach has not been adopted by μCT device manufacturers.^11^ Thus, voxel size (nominal resolution) should be reported, preferably in units of microns (μm).

### Phantom calibration for BMD

For μCT scans reporting BMD, it is recommended to use a minimum of two calcium hydroxyapatite (CaHA) phantoms, ranging from 2-4 mm (diameter) and densities of 0.25 and 0.75 g.cm^-3^ for routine calibrations. Phantoms should be scanned and reconstructed using the same parameters as fish specimens. BMD in fish should be reported in g.cm^-3^ or mg.cm^-3^ of HA.

For 2D X-rays, thus far only relative attenuation has been used to compare bone densities across specimens. Aluminum wedges can be used for phantom calibration; however, a disadvantage is that the density of the radiographic image is calibrated in millimeters of aluminum equivalent thickness.^31^ Due to the limitations of 2D imaging, areal BMD (aBMD) values will always be relative and not absolute. The calibration used for measuring bone density from 2D X-rays and 3D μCTs should be reported in Methods.

### Image quality

Scanning artifacts are commonly found in μCT and may prevent accurate results. After scanning, images should be carefully inspected visually. Two common artifacts are motion artifacts and beam hardening.^11^

### Motion artifact

Maintaining the sample in a fixed position within the sample holder is critical to minimize blurring and double images, as well as long-range streaks. To minimize motion artifacts, often resulted from sample sliding and tissue dehydration during long scans, we recommend ensuring the sample is stabilized and hydrated using the mounting methods described above.

### Beam hardening artifact

Occur when the X-ray beam encounters differences in absorption from different angles and along different paths through the object, due to the very dense object itself or its parts. For multiplexed scanning, we recommend equidistant fish positioning from the scan center to minimize beam hardening artifacts.^6^

## Recommended parameters for image segmentation and thresholding

Here, we explain fundamental parameters for image segmentation and thresholding, and recommend those to be reported (Table 2).

### Segmentation

**Figure 2:**
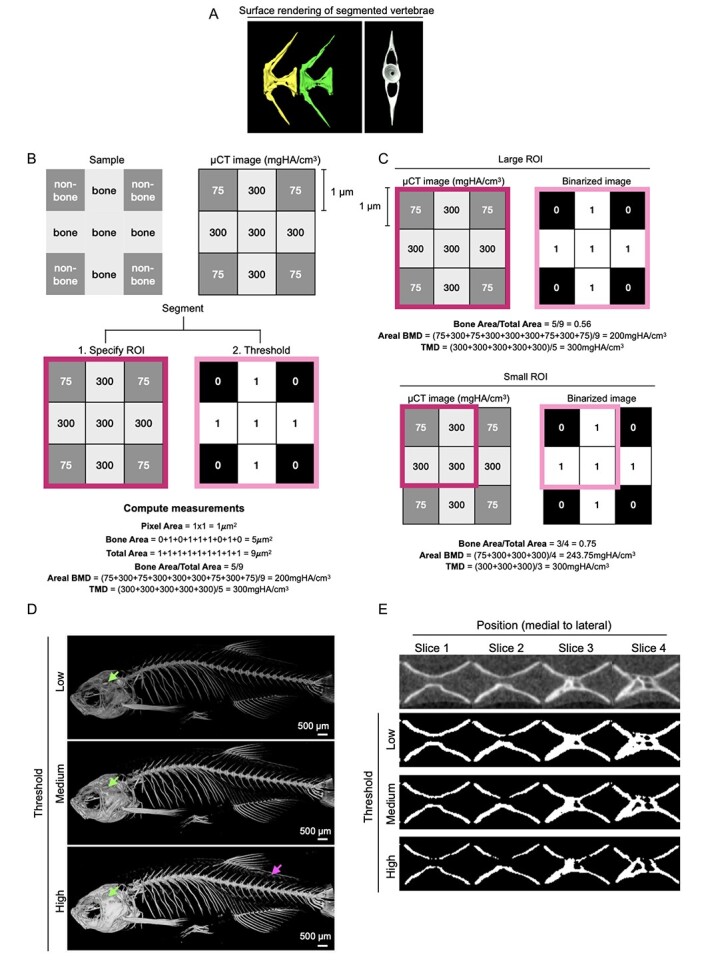
Imaging processing: Segmentation, effects of ROI on μCT measures and thresholding. (A) Example of segmented vertebrae from 3-month-old zebrafish visualized with surface rendering (yellow and green in a lateral view, and gray in a cross-section view) using Amira (FEI). Image as acquired with a 10 μm voxel size. (B) Principles of μCT image analysis from segmented regions. On the left a box representing a sample containing bone and non-bone, on the right the respective hydroxyapatite concentrations in a 3 × 3 pixel image. To segment the region, an ROI (magenta box) is selected (1) and a threshold applied (pink box) (2) to identify pixels containing bone (1) and non-bone (0) represented within a binarized image. Note that BMD is computed as the average attenuation-equivalent hydroxyapatite concentration for all pixels within the ROI including bone and non-bone pixels. In contrast, TMD is computed as the average attenuation-equivalent hydroxyapatite concentration using only bone pixels within the ROI. (C) The importance of reporting ROIs. Measures are computed using a large (3 × 3) and a small (2 × 2) ROI. Note that bone area/total area and areal BMD are different using the two different ROIs, but TMD remains constant. (D) Volume rendering after applying different thresholds. Note that more bones are seen at high threshold levels. Such as that the sphenoid bone covering the otolith (green arrow) and a few scales (magenta arrow) are seen in the example only at high threshold. Amira (FEI) was used to process images. Image is from a 1-yr-old zebrafish and was acquired with a 21 μm voxel size. Scale bars = 500 μm. (E) Segmentation arising from different thresholds in a zebrafish vertebra. Top row shows four different μCT image slices of the same vertebra (v5) in an adult zebrafish. Slices are in the sagittal view, with slice 1 the most medial, and slice 4 the most lateral. Bottom three rows depict binarized images following segmentation using a low, medium, and high threshold. In this example, no single threshold appears to result in a segmentation that perfectly correlates with bone voxels identifiable by eye for all four slices. It is suggested that when choosing a threshold manually, multiple samples and locations within the image be examined. Image was acquired with a 10.5 μm voxel size.

Image segmentation refers to the partitioning of a digital image into image segments, such as single vertebrae (Figure 2A). Most commonly, 2D X-ray and 3D μCT image segmentation for bone microstructure and density analysis involves defining a region of interest (ROI) and thresholding (Figure 2B). The primary purpose of ROIs is to isolate specific regions within a μCT image, such as a single bone amid the rest of the skeletal structure, or a particular compartment within a bone, which can then be thresholded to isolate bone voxels from non-bone voxels. However, it is possible to perform segmentation of bone voxels without thresholding. This can be performed, for example, by manually contouring the bone of interest in an unthresholded image. However, this method becomes increasingly difficult for bones with complex shapes and will be prone to inter-individual variability in how readers classify bone vs soft tissue. Whether segmentation was performed by defining an ROI and thresholding, or contouring bones in unthresholded images, should be specified.

ROIs can be established through manual delineation of contours, performed slice by slice, or by creating a defined geometric shape (eg, a box or cylinder) surrounding the structure of interest. After segmentation, ROIs will consist of voxels containing bone and voxels containing soft tissue (Figure 2B). Notably, employing different ROIs for the same skeletal object may yield disparate morphometric or densitometric values if the calculation depends on the value of the volume of the ROI itself (Figure 2C). For example, when two ROIs of varying volumes are applied to the same skeletal object, BMD will register as higher for the smaller volume ROI. This discrepancy arises because ROI volume serves as the denominator in the calculation of volumetric BMD. To ensure meaningful comparisons of data across diverse studies, it is imperative to provide details about whether the ROI was generated through the manual delineation of contours or by defining a geometric shape (eg, a box or cylinder), including any reference to landmarks employed in ROI specification.

### Thresholding

Thresholding is used to partition voxels into those that comprise bone vs those that do not. Thresholding is typically performed on a structure within an ROI (Figure 2D and E). The selection of an appropriate threshold is critical for accurate analysis (ie, BMD, TMD, etc.). The most straightforward method is a manually selected global threshold applied to all images in the experiment. It ensures that any observed differences between the control and experimental groups are not an artifact of different thresholds. Thresholds can be manually chosen by qualitatively assessing whether the segmented voxels adequately recreate the presumed bone morphology. In this case, the selected global threshold should be reported in units of g HA/cm^3^ or mg HA/cm^3^.

Global thresholds may not be suitable in several cases. The zebrafish undergoes continuous mineralization and growth through the life course, and thus appropriate thresholds might vary across different developmental stages. The developmental progress of zebrafish also varies within the same group of embryos,^32^ which is influenced by the fish’s standard length. Additionally, genetic mutations can substantially modify TMD, making a global threshold less applicable. To address these complexities, previous research has employed specimen-specific thresholds determined through semi-automatic image threshold algorithms when analyzing adult zebrafish.^6^ For semi-automatic threshold, it is crucial to provide comprehensive information about the image threshold algorithm and the steps involved in image pre-processing to enhance the clarity and reproducibility of the data.

## Key parameters for image analysis

Next, we discuss bone density and morphometry, recommending key parameters to be reported (Table 2).

### Bone density

It is important to define two distinct measures of bone density: (1) “BMD,” which measures attenuation-equivalent hydroxyapatite concentration within a volume that contains a mixture of bone and soft tissue, and (2) “TMD,” which measures attenuation-equivalent CaHA concentration within the volume of calcified bone tissue, excluding soft tissue. The differences between BMD and TMD have important ramifications about their interpretation. For instance, BMD provides information about the quantity of bone within the volume being analyzed, as well as the material density (Figure 2B). In contrast, TMD provides information about the material density of the bone itself, but not about the quantity of bone (Figure 2B). There are also important methodological differences in their computation. For instance, the computation of TMD requires specifying segmented bone voxels (eg, by drawing contours and thresholding), as these are needed to compute the attenuation-equivalent hydroxyapatite concentration within the bone voxels only. In contrast, the computation of BMD does not require specifying segmented bone voxels. This is because BMD measures attenuation-equivalent CaHA concentration within a mixture of bone and soft tissue and solely requires specifying an ROI to define a volume of unthresholded pixels. Owing to the important differences in BMD and TMD, when reporting results, it should be clear whether BMD or TMD was examined.

As described above, the size and shape of the volume of interest will determine the numerator of the BMD calculation and thus greatly influence the resulting BMD values. The same holds true for BV/TV, that is, the ratio of bone volume to total volume. In this case, TV is calculated as the total volume of the ROI, and thus its size and shape will alter the denominator in the BV/TV calculation. As such, it is critical that when reporting BMD or BV/TV, a description of the volumes of interest, including any relevant landmarks, should be provided with sufficient details for readers to replicate the analyses.

It is worthwhile to note the influence of sample hydration on BMD measurement. If a sample is removed from alcohol, this will cause dehydration particularly in the soft tissue which can introduce air bubbles into the sample. Because BMD measures attenuation-equivalent hydroxyapatite concentration within the soft tissue, this will result in severely compromised BMD values, and negative values are even possible.

### Bone morphometry

Here, we highlight general recommendations for morphometric analyses. These methods fall into two categories: landmark- and segmentation-based approaches. Landmark-based methods involve placing landmarks on detectable anatomical features to compute distances, areas, or angles.^6^^,^^7^ Landmark-based methods do not require segmentation and are useful for analyzing complex bones like craniofacial bones. Landmarking can be done manually by identifying anatomical features from maximum intensity projections using software such as ImageJ/FIJI (2D) or Image Slicer (3D). Landmarking can be done automatically using atlas-based methods.^33^ Detailed descriptions of landmarking procedures should be provided, including lists or schematics of anatomical features where landmarks were placed, calculations performed, and the method used for landmark placement (eg, semi-automatic or manual placement).

Segmentation-based methods compute morphometric descriptors using segmented voxels, which, as described above, are either obtained via ROI specification and thresholding, or directly contouring a bone of interest. Commercial μCT systems usually come with analysis software that computes such measures, which include volume (computed by summing the volume of voxels classified to bone), thickness (which can be computed, for example, by calculating locally the largest possible spheres that fit into a geometry and then averaging this quantity for the entire bone), and surface area.^6^^,^^28^ When using segmentation-based methods, in addition to the details on segmentation and thresholding described above, a description of the methods used for quantification of measures should be provided. Details should include the functions and software (name and version) used for calculation (eg, “bone thickness was computed using the Thickness function implemented in the BoneJ plugin in ImageJ”).

Measures to report should be chosen carefully. For instance, commercial μCT systems also come with analysis software that compute measures to assess trabecular bone, which is much more prevalent in the skeletons of small rodents compared to zebrafish. These include trabecular number, trabecular separation, “connectivity density,” and “structure model index.” In general, when the region being analyzed contains compact bone—which is almost always the case in zebrafish—the use of these measures is not recommended due to the difficulty in their interpretation.

**Figure 3:**
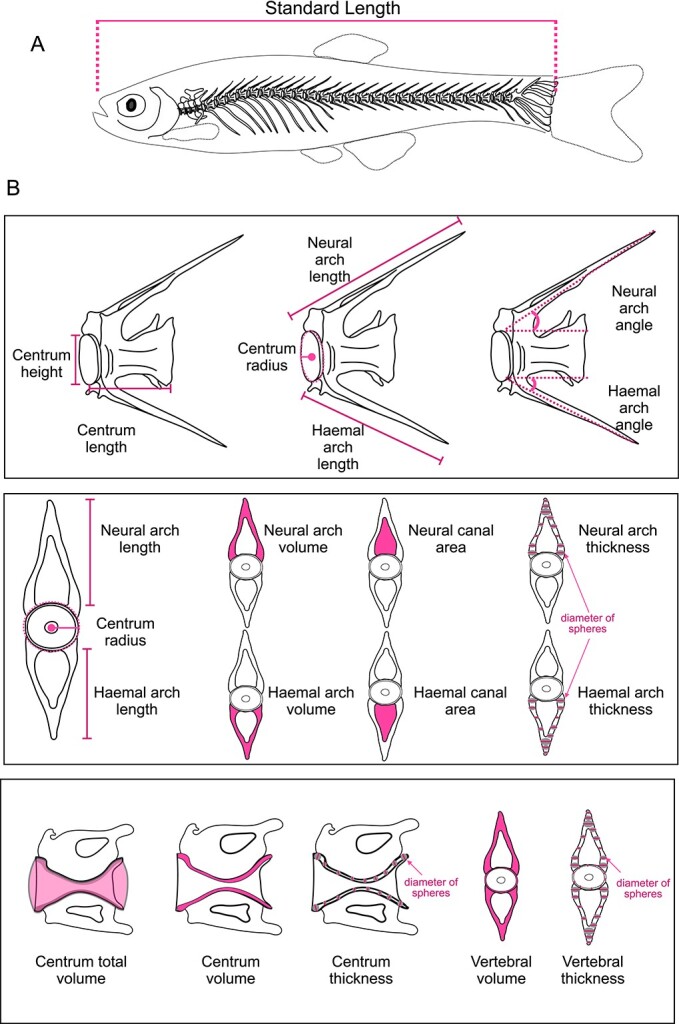
Diagrammatic view of the skeletal anatomy of zebrafish focusing on routine morphometry measurements. (A) Standard length taken from lateral view of a fish. (B) Measurements taken from μCT images. The description for each measurement is provided in Table 3.

It is understood that each publication brings forth distinct inquiries and necessitates a variety of morphological analyses to respond to these inquiries. In Figure 3 and Table 3, we provide common morphometric measurements derived from 3D images.

**Table 3:** Common measures for bone morphometry in the adult zebrafish spine from μCT.

Measurement	Description	Unit	Abbreviation
**Standard length**	Length from the tip of the snout to the base of the tail	mm	SL
**Centrum length**	Length of the centrum along the cranial-caudal axis as measured by the length between opposite endplates	μm	CL
**Centrum height**	Height of the centrum along the dorsal-ventral axis as measured by the length from the most dorsal point of the centrum to the most ventral point at the endplate region	μm	CH
**Centrum radius**	Radius of the centrum at the endplate as measured by the length from the center of notochord canal to the endplate, can also be approximated as 0.5*centrum height	μm	CR
**Centrum thickness**	Average centrum bone thickness, typically computed by defining the diameter of the greatest sphere that fits within the structure at each point, and averaging this value for the entire structure	μm^2^	C.Th
**Centrum volume**	Volume of the centrum bone only, typically computed by summing the volume of voxels comprising the structure	μm^3^	CV
**Centrum total volume**	Volume of the centrum including soft tissue and surrounding bone	μm^3^	CTV
**Neural arch length**	Length from the base of the neural arch at the attachment site of the centrum to the most dorsal point of the neural spine	μm	Na.L
**Neural arch thickness**	Average neural arch bone thickness, typically computed by defining the diameter of the greatest sphere that fits within the structure at each point, and averaging this value for the entire structure	μm	Na.Th
**Neural arch volume**	Volume of the neural arch bone only, typically computed by summing the volume of voxels comprising the structure	μm^3^	Na.V
**Neural arch angle**	Angle between two-line segments: (i) the line connecting the base of the neural arch at the attachment site of the centrum to the most dorsal point of the neural spine and (ii) the line connecting the opposite centrum endplates along the cranial-caudal axis	degrees (°)	Na.Ang
**Neural canal area**	Area of the neural canal, typically computed by specifying a polygon ROI within the canal and determining its area	μm^2^	Nc.A
**Haemal arch length**	Length from the base of the haemal arch at the attachment site of the centrum to the most ventral point of the haemal spine	μm	Ha.L
**Haemal arch thickness**	Average haemal arch bone thickness, typically computed by defining the diameter of the greatest sphere that fits within the structure at each point, and averaging this value for the entire structure	μm	Ha.Th
**Haemal arch volume**	Volume of the haemal arch bone only, typically computed by summing the volume of voxels comprising the structure	μm^3^	Ha.V
**Haemal arch angle**	Angle between two-line segments: (i) the line connecting the base of the haemal arch at the attachment site of the centrum to the most ventral point of the haemal spine and (ii) the line connecting the opposite centrum endplates along the cranial-caudal axis	degrees (°)	Ha.Ang
**Haemal canal area**	Area of the haemal canal, typically computed by specifying a polygon ROI within the canal and determining its area	μm^2^	Ha.A
**Vertebral thickness**	Average bone thickness in the entire vertebra, including the centrum, neural arch, and haemal arch. Typically computed by defining the diameter of the greatest sphere that fits within the structure at each point, and averaging this value for the entire structure	μm	V.Th
**Vertebral volume**	Volume of bone in the entire vertebrae including the centrum, neural arch, and haemal arch. Typically computed by summing the volume of voxels comprising the structure. Can be approximated by computing the sum of the neural arch, centrum, and haemal arch volumes	μm^3^	VV

## Anatomical sites for analysis and nomenclature

Bone morphometry and density analysis can be performed at several different anatomical sties. Here, we provide a discussion of zebrafish skeletal anatomy for regions of the axial skeleton, vertebral elements, and skull. We recommend using the anatomical nomenclature described here when reporting X-ray/μCT results.

### Axial skeleton

**Figure 4:**
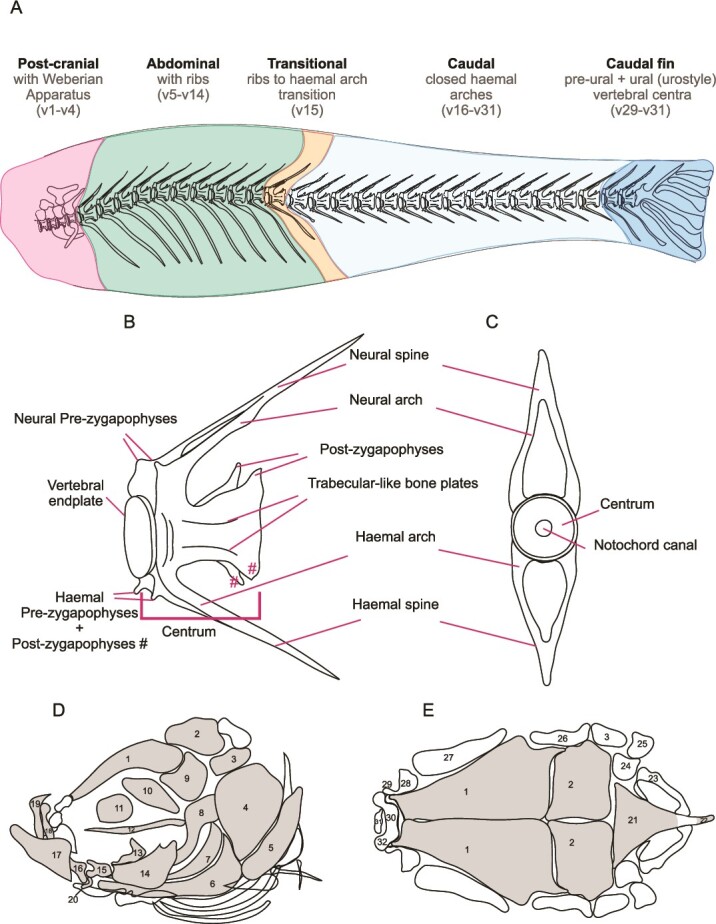
Schematic view of the mineralized elements of the adult zebrafish vertebral column and skull that can be recognized on μCT images. (A) Vertebral column. The different regions are indicated with colors. Pink, postcranial vertebrae with modified arches and processes that connect the swim-bladder with the inner ear. Green, abdominal vertebrae carry ribs. Orange, transitional vertebrae, the transformation between rib bearing and haemal arch bearing vertebrae. In zebrafish often typically one vertebral body. Light blue, caudal vertebrae with closed haemal arches. Dark blue, caudal complex. The caudal complex comprises the elements of the endoskeletal caudal fin support. This is the urostyle, a fusion product of several vertebral centra and the preeural vertebrae that also show a variable tendency to fuse. Dermal fin rays and the endoskeletal elements of pectoral, pelvic, dorsal, and anal fins are not shown. Likewise intramuscular bones are not shown. (B and C) Vertebral body and spines. (D and E) Schematic view of the zebrafish skull. (D) Lateral view with elements of the neural and the splanchnocranium. (E) Dorsal view of the neurocranium. Gray regions: bones easily visualized from μCT scans. 1-frontal, 2-parietal, 3-pterotic bone, 4-opercle, 5-sub-opercular, 6-interopercular, 7-pre-opercular, 8-hyomandibular, 9-sphenoid, 10-pterosphenoid, 11-orbitosphenoid, 12-parasphenoid, 13-ectopterigoid, 14-metapterigoid, 15-quadrate, 16-anguloarticular, 17-dentary, 18-maxillary, 19-pre-maxillary, 20-retroarticular, 21-supraoccipital, 22-basioccipital, 23-exoccipital, 24-epioccipital, 25-post-temporal, 26-sphenotic, 27-supra-orbital, 28-lateral ethmoid, 29-nasal, 30-ethmoid, 31-kin-ethmoid, 32-pre-ethmoid. Nomenclature after Bird and Mabee,^36^ Cubbage and Mabee,^37^ Bensimon-Brito *et al.*^58^

The post-cranial axial skeleton includes the vertebral column and associated caudal fins. The vertebral column is the major skeletal component in zebrafish, fully formed around 2 mo post-fertilization and similar to mammals, comprised of vertebral bodies separated by intervertebral discs; however, it is mostly composed of cortical bone as each vertebra shows only a few trabeculations surrounding the vertebrae.^9^^,^^34^^,^^35^ Zebrafish vertebrae are formed by intramembranous ossification and contain blood vessels and fat instead of hematopoietic tissue.^2^ The vertebral column is the most studied skeletal region for BMD measurements in fish modeling bone mass disorders. The vertebral column is regionalized into postcranial/Weberian, abdominal, transitional, caudal, and caudal fin supporting vertebrae (Figure 4A). The postcranial vertebrae comprise vertebrae 1-4 (v1-v4) as part of the Weberian apparatus. The rib-bearing abdominal vertebrae (referred to as precaudal in some literature), usually encompassing 10 vertebrae (depicted as v5-v14). Generally, only one vertebra (v15) is present in the zebrafish transitional region, characterized by elongated unfused haemal arches and shortened ribs. The caudal region is usually comprised of 14 vertebrae (v16-29). The most posterior caudal fin vertebrae (variable numbers, v29-v31) are modified to support the caudal fin. Note that the number of vertebrae in wildtype zebrafish can vary; Bird and Mabee found that the modal number of vertebrae is 31: 4 Weberian, 10 precaudal, 14 caudal, and 3 caudal fin vertebrae.^36^

### Vertebral elements

Characteristically of vertebral elements, abdominal vertebrae have neural arches and ribs; the transitional vertebra has an elongated haemal arch and shortened ribs; and the caudal vertebrae have neural arches and haemal arches (Figure 4B and C). Additionally, each vertebra possesses a vertebral body, which comprises multiple elements/regions, including a centrum, vertebral endplates, and vertebral bony plates (Figure 4B and C). The bony plates, which are more pronounced in adult fish greater than 1 yr old, are in the periphery of the centrum.

### Skull

The zebrafish skull comprises a complex arrangement of 74 bones,^37^ contrary to 22 in humans. Similarly to humans, zebrafish have conserved anatomical organization of the skull vault, formed with cranial plates and sutures. However, the sutures in zebrafish overlap throughout life and never fuse.^38–40^ Also unlike mammals, the cartilage in the zebrafish craniofacial skeleton can regenerate after injury, as demonstrated with jaw ligament injury and jaw injury^41^^,^^42^ In Figure 4D and E, we have labeled skull bones that can be readily visualized from μCT images and labeled them based on previous anatomic descriptions^37^^,^^38^ and segmentations from μCT data.^43^

## Other recommended parameters when reporting results

Other recommended indices for reporting are presented in Table 2**.**

### Reporting age and standard length

Despite zebrafish lifelong growth, with concomitant changes in bone size, shape, and mineral density, their skeleton ages as in mammals. Zebrafish skeletons show signs of structure decay (reduced bone quantity and quality with increased bone fragility).^8^^,^^44^ We recommend that BMD calculations can be first obtained in 3-mo-old fish, and aging studies should be performed in fish over 2.5 yr, when skeletal degeneration has been detected.^8^ Environmental conditions have a strong influence on developmental progress in zebrafish. Developmental progress is significantly more correlated with standard length than with age in postembryonic animals.^32^ However, both standard length and age exhibited relatively high correlations.^32^ The reporting of both measures can aid in staging, for example, in cases where experimental treatments alter growth and, by extension, standard length. Thus, it is recommended that the standard length of the animals used for the study be reported along with age.

### Reporting strain and sex

Charles *et al.*^9^ analyzed over 200 zebrafish of different ages, strains, and sexes to study variation within bones using μCT. Comparison of measurements derived from wild-type male and female fish of ages varying from 3- to 10-mo-old revealed that BMD measurements highly depend on the background strain.^9^ Thus, when reporting results, strain should be specified. The studies of Charles *et al.* did not reveal significant sex differences in the measures that they analyzed. Nevertheless, we recommend reporting whether studies were performed in male, female, or mixed-sex groups.

### Controls: use and reporting

We recommend that when investigating effects of mutations or chemical treatments, clutch mates be used for controls. This will help to minimize confounding effects arising from genetic and environmental differences in clutches raised at different times. When reporting results, details of the controls (eg, clutch mates) used in the experiments should be specified.

### Data normalization

Several approaches have been used to normalize μCT data to account for differences in developmental progress in zebrafish. A common approach is to divide the μCT measure being analyzed by the standard length (SL), which will be most effective when there is a positive linear correlation. Charles *et al.* examined several μCT measures in zebrafish and found that most, but not all, were positively correlated with SL.^9^ One exception was neural arch angle, which was inversely related to SL, and thus not appropriate to be normalized by dividing by SL.^9^ When μCT measures have a nonlinear relationship with SL, other normalization schemes may be more optimal. Hur *et al.*^6^ showed that the dependence of μCT measures on SL can be described as a power law. As such, dividing y by SL^α^, where α is a scaling exponent, may be a more appropriate and effective normalization compared to dividing *y* by SL, particularly when α > 1.

One limitation of the normalization schemes described above is that when comparing mutants to controls. As noted by Charles *et al.*, “analysis of mutants with skeletal deformities raised an interesting caveat to normalization by length. In mutants affecting skeletal growth, body proportion is frequently altered, leading to a ‘stumpy’ phenotype. In these cases, standard length is no longer an independent measure of staging between fish.^9^” In such cases, it has been suggested to use orbital diameter as an alternate measure for normalization^9^ or power law scaling.^6^ Although several approaches for normalization can be pursued, the method used should be described in detail and clearly discussed.

### Presentation of images

For μCT visualization, options include displaying individual slices and generating max intensity and mean intensity projections. Max intensity projections show the maximum TMD through the stacks, useful for visualizing TMD differences. Mean intensity projections resemble 2D X-rays, allowing visualization of structures like the swim bladder not visible in max intensity projections.^7^ Additional visualization methods include creating 3D models and surface renderings from segmented data, which can be generated using either software supplied by the manufacturers of commercial μCT systems, or free open source software (https://www.slicer.org).^45^ Surface renderings, while visually appealing, may mask TMD alterations. When presenting images, specify the type (eg, max intensity projection, surface rendering) and the methods used for image generation. Orient images with the anterior end on the left and dorsal side up for consistency.

## Complementary approaches

**Figure 5:**
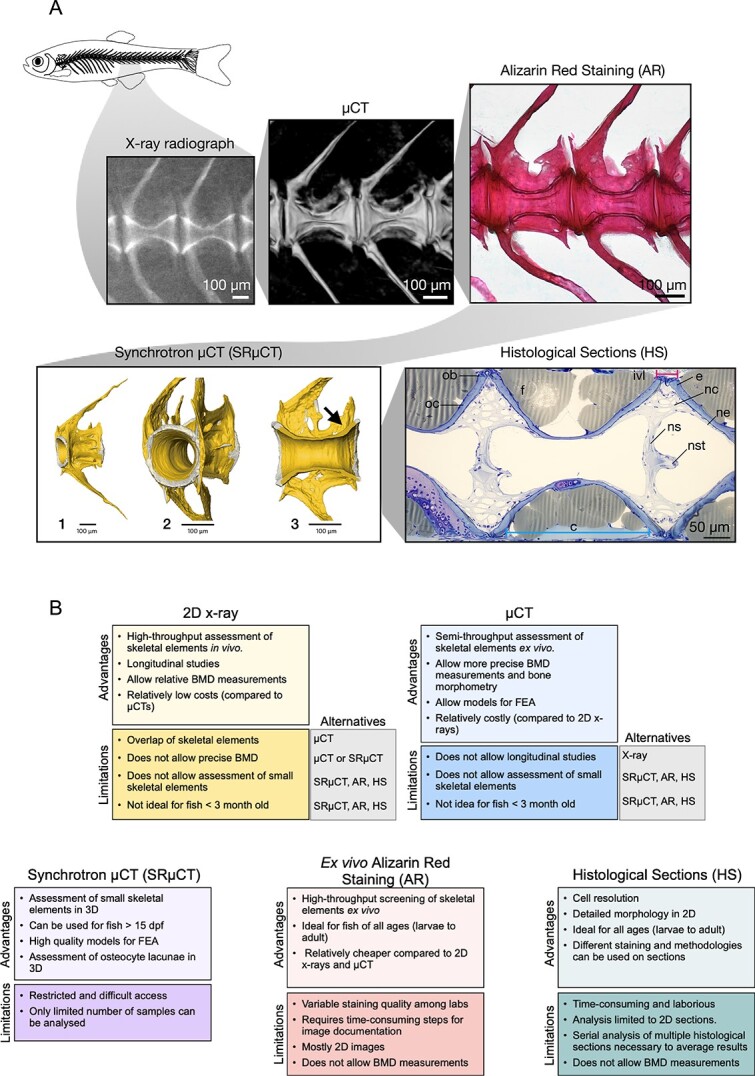
Beyond X-ray radiographs and μCT: complementary approaches. (A) A close look at the caudal vertebrae of a 3-mo-old zebrafish using X-ray radiograph, μCT, alizarin red staining (AR), synchrotron μCT (SRμCT) and histological sections (HS) stained with toluidine blue. Note that each technique allows to capture different depth of morphological details of the vertebra. SRμCT shows a vertebra with arches (1), vertebrae zoomed in (2) and a cross section through the vertebra (3). Note that non-mineralized bones can be detected (arrow, gray area of the endplates). (B) Advantages and limitations of X-ray radiographs, μCT, and complementary methodologies. FEA (finite element analysis). c-centrum (blue line), e-endplate; f-fat; ivl-intervertebral ligament (magenta line); nc-notochord cells; ne-notochord epithelium; ns-notochord septum; nst-notochord strand; Ob-osteoblast; oc-osteocyte. Scale bars = 100 μm.

Here, we discuss techniques that can be used to supplement X-ray radiography and μCT-derived morphometric and densitometric analysis (Figure 5). The choice between techniques depends on the specific requirements of each study, such as the desired resolution, sample size, and the type of features that need to be visualized. In the future, it can be expected that new generations of desktop devices for microcomputed tomography or nanocomputed tomography (ie, nanoCT) will be able to resolve even the smallest skeletal structures with high information depth.

### 3D X-ray microscopy

3D X-ray microscopy uses X-ray optics to achieve optical magnification, enabling high-resolution imaging even for small fields of view.^26^^,^^28^ Therefore, the visibility of features with low X-ray absorption contrast, such as soft tissues or small-scale bone structures as osteocyte lacunae, can be resolved.^1–3^

### Synchrotron radiation micro-computed tomography (SRμCT)

SRμCT provides a high-intensity and monochromatic X-ray beam, allowing the acquisition of quantitative, high-resolution 3D images with a high signal-to-noise ratio. Zebrafish bones scanned using SRμCT can reach an isotropic resolution of 0.3 μm, allowing to analyze bone and soft tissues comparable to histological analyses, with the detection of osteocyte lacunae.^8^^,^^16^^,^^29^^,^^46^ However, there are only a few synchrotrons in the world, which poses an imperative limitation for their routine use and accountability.

### Whole-mount alizarin red and Alcian blue staining

Alizarin red staining offers a quick and cost-effective method for analyzing numerous specimens. With high-power stereo or compound microscopes, whole-mount staining provides resolution surpassing μCT scans, allowing visualization of non-mineralized bone and osteoid.^16^ Classical staining protocols typically use Alizarin red S for mineralized bone and Alcian blue 8GX for cartilage.^47^ However, the acidic nature of Alcian blue can remove minerals from early mineralizing structures, leading to false-negative results. To address this, Walker and Kimmel^48^ proposed an acid-free double staining protocol suitable for early skeletal development in zebrafish. To enhance visualization of weakly stained bones, fluorescent light can be used, as Alizarin red is highly fluorescent.^49–51^ Imaging or digitizing may be necessary for analyzing large specimen numbers in detail.^52^ While it allows visualization of mineralized structures, quantitative assessment of mineralization or density is not feasible. The staining fades over time, and staining intensity does not correlate with mineralization degree. Early mineralization stages exhibit intense staining due to available calcium, but once minerals settle into apatite, staining intensity diminishes.^53^

### Histological sections

Zebrafish cells are half the size of human cells, and bone trabeculae width can be <1 μm.^54^ Thus, tissue embedding in epoxy resin for superior-quality thin sections is desirable (1 μm). Further, specimens can be used for transmission electron microscopy.^55^ Other resins are glycol methacrylate (GMA) and methyl methacrylate (MMA). GMA provides enzyme detection, the study of fluorescent reporter lines, and the visualization of *in situ* hybridization.^16^^,^^56^^,^^57^ MMA embedded material can be used for a range of staining protocols. This includes mineral detection and immunohistochemistry, after resin removal. We recommend the use of resin embedding for routine histology if possible and only use paraffin or cryotome sections as an exception.^52^^,^^55^

## Supplementary Material

JBMR_Supplementary_table_1_final_zjae171
