# Supplementary material for: Standardization of bone morphometry and mineral density assessments in zebrafish and other small laboratory fishes using X-ray radiography and micro-computed tomography
Source: J Bone Miner Res. 2024 Oct 30;39(12):1695–710. doi: 10.1093/jbmr/zjae171 (PMC11642618; doi:10.1093/jbmr/zjae171)
Supplement: JBMR_Supplementary_table_1_final_zjae171 [file jbmr_supplementary_table_1_final_zjae171.docx]

**Supplementary table 1.** List of reported parameters for µCT in fish from the literature

| **Author/year** | **Species** | **μCT machine/ phantom /acquisition** | **Scanning parameters reported** | **Strain/age, sex** | **Skeletal region analysed (software)** | **Measurements** |
| --- | --- | --- | --- | --- | --- | --- |
| (Siccardi et al., 2010) | *Danio rerio* (zebrafish) | μCT 40 (Scanco Medical)  phantom of five cylinders (Al) | 70 Kilovoltage peak (kVp); 114 mA; integration time 200 ms | wild-type; 16-week-old | Whole body (WB)  Spinal column | BMD (mgHA/cm^3^)  Bone volume (mm^3^)  Bone density/bone volume |
| (Hur et al., 2017) | *Danio rerio* (zebrafish) | vivaCT 40 (Scanco Medical) | 21μm voxel size: 55kVp, 145μA, 500proj/180°, 200ms integration time.  10.5μm voxel size: 55kVp, 145μA, 1000proj/180°, 200ms integration time | ARO and AB (wild-type);  *bmp1a*^sa2416^, *plod2*^sa1768^; mixed sex, age not reported | whole body  precaudal and caudal vertebrae  (FishCuT) | neural arch (Neur), centrum (Cent), haemal arch/ribs (Haem) for : Tissue Mineral Density (Tot.TMD, Cent.TMD, Haem.TMD, and Neur.TMD; mgHA/cm^3^), Volume (Tot.Vol, μm^3^), Thickness (Th, μm), Surface Area (SA, μm^2^), centrum length (Cent.Le, μm) & diameter; intra-specimen variation (TMD.sd and Th.sd) |
| (Charles et al., 2017) | *Danio rerio* (zebrafish) | μCT 35 (Scanco Medical)  HA calibration phantoms  global threshold for bone/non-bone cut-off at 452.7 mgHA/cm^3^ | 6 μm voxel size; X-ray tube potential of 55 kVp, 0.5 mm Al filter, X-ray intensity of 0.145 mA & integration time 600 ms per slice (vertebrae) and 800 ms (parasphenoid) | bmp1a/weld^edt31169^ and csfr1^amh5^; male and female, at 3, 5, 9, and 10 -month-old | C1 and C2 vertebrae and parasphenoid | Bone density of the centra (BMD; mgHA/mm^3^) and bone volume to tissue volume (BV/TV); radius of the vertebral opening  Width & height of the neural arch; arch area  Parasphenoid: BV/TV |
| (Charles et al., 2017) | *Danio rerio* (zebrafish) | Skyscan 1173 (Bruker)  (silver nitrate incubation) | 7.14μm voxel size, source voltage 70 kV, 80 μA current; exposure time of 1500 ms; over 240°, 0.2° rotational step | Juvenile (13mm standard length) |  | Morphology |
| (Suniaga et al., 2018) | *Danio rerio* (zebrafish) | Skyscan 1272 (Bruker) | 1µm voxel size  45 kV and 200 μA without filter | 4.5-month-old, strain and sex not reported | 9 vertebral bodies | tissue volume (TV)  bone volume (BV)  vertebral length (VL)  “autocentrum volume” |
| (Monma et al., 2019) | *Danio rerio* (zebrafish) | System R_mCT (Rigaku, Tokyo, Japan)  HA phantoms: range 200–1550 mg/cm^3^ | 10 μm voxel size; Voltage 90 kV; current 150 μA; magnification 20×; slice thickness (scanning width) 10 μm; exposure time 2 min | AB wild-type; 3, 6, 9, 12 and some 45-month-old;  male and female | first caudal vertebra  (TRI/3D-BON software) | BMD  bone mineral content (BMC); BV, TV  trabecular bone volume fraction (BV/TV)  Trabecular thickness (Tb.Th)  trabecular number (Tb.N)  Tissue space star volume (Vol*m.space; mm^3^])  Trabecular star volume (Vol*tr) [mm^3^] |
| (Marcé-Nogué and Liu, 2020) | *Danio rerio* (zebrafish) | Xradia VersaXRM-520  and Skyscan 1276 (Bruker) | Xradia: 4.64 μm voxel size: 100kV, 9 W, 1601projections 4s/exposure.  Skyscan/Bruker: 5.05 μm voxel size: 55kV, 200 μA, 900 images/ 675ms/exposure | adult, sex not reported | Weberian ossicles: claustrum, scaphium, intercalarium, and tripus | length of Weberian chain;  shape variables  Finite Element Analysis (FEA) |
| (Waldmann et al., 2021) | *Danio rerio* (zebrafish) | Skyscan 1172 (Bruker) | 5.43 μm voxel size, voltage of 60 kV, current of 167 μA, | Wild-type and mutant (*nkx3*.*2*^uu2803/uu2803^ (3-month-old), sex not reported | premaxilla and maxilla; basioccipital and exoccipital | Bone fusion  Morphology of the Weberian apparatus |
| (Kague et al., 2021) | *Danio rerio* (zebrafish) | XT H 225ST μCT scanner (Nikon)  CaHA phantoms (0.25 and 0.75 g/cm^3^) | 5 µm and 21 µm voxel size; X-ray source of 130 kV, 53 µA, no additional filters | 1, 2 and 3 year-old wild-type; ctsk crispant (1 year old); sp7^hu2790^ | Caudal vertebrae | spinal morphology;  TMD; centrae length, centrae volume, intervertebral disk calcification |
| (Watson et al., 2021) | *Danio rerio* (zebrafish) | vivaCT 40 (Scanco) | 21 µm voxel size: 55kVp, 145mA, 500proj/180 ̊, 200 ms integration time | *tspan12, ing3, cped1, fam3c; wnt16 crispants* (3-month-old) | 20 anterior-most pre-caudal and caudal vertebrae  (FishCuT) | As above (Hur et al., 2017) |
| (Hino et al., 2024) | *Danio rerio* (zebrafish) | Skyscan 1172 (Bruker) | 35 kV, at resolution of 2.5 μm/pixel | Tg(*osx:col1a2-gfp*) and wild-type siblings; | whole body | average mineral intensity at 5 vertebrae segments |
| (Kaliya-Perumal et al., 2024) | *Danio rerio* (zebrafish) | Skyscan 1173 (Bruker) | not stated | *kcnk5^bdt30mh/+^ & il11ra^bns251^* and wild-type siblings | pectoral fins incubated with 1% silver nitrate  (AMIRA & 3D slicer) | Bone volume |
| (Raman et al., 2024) | *Danio rerio* (zebrafish) | Skyscan 1272  (Bruker) | WB: 70 kV and 100 μA, voxel size 21 μm, 0.5 mm Al filter; first precaudal vertebrae: isotropic voxel size = 7 μm | *col10a1a^−/−^* and *fbln1^−/−^* and wild-type siblings; 1 y old, sex not reported | whole-body and precaudal scans | 21 μm voxel size: FishCuT Software v. 1.2;  7μm voxel size: precaudal vert. 6–8, vertebral thickness and vertebral length |
| (Ramli et al., 2023) | *Danio rerio* (zebrafish) | SkyScan 1172 (Bruker) | 50 kV, WB: resolution 12 μm/pixel; 5 μm/pixel for abdominal or caudal region | *piezo1^−/−^ ; piezo1^11aa^* ^del/11aa del^ and wild-type siblings; 3-4 mpf; both sexes | whole-body scans | TMD;  Morphology: IVD calcification, bone fusion, ectopic bone calcification, end-plate sclerosis, and osteophytes |
| (Ofer et al., 2019) | *Oryzias*latipes (medaka) | SkyScan 1172  (Bruker) | 50 kV and 200 μA; 4,000 projections for each scan; angular range of 360°, isotropic voxel size=2μm, exposure time=3.5 sec | Young adult (8–12-month-old); males | caudal vertebra | FEA model |
| (Butylina et al., 2022) | *Nothobranchius furzeri* (killifish) | μCT 35 (Scanco Medical) | 6 μm voxel size; 70 kVp, 114 μA, and energy 8 W | male and female between 5 and 20-week-old | whole-body scans | BMD;  cortical thickness (Ct.Th), bone volume (BV), and height of the vertebral bodies |
| (Abe et al., 2024) | *Nothobranchius furzeri* (killifish) | ScanXmate-S090R  (Comscan, Japan) | voltage 45 kV; current 180 μA;  magnification ×4.9; brass filter | GRZ (GRZ-AD) strain; 9 “old” (precise age not reported), males | (TRI/3D-BON software) | nine caudal vertebrae: BV, BMD |
| (Cho et al., 2023) | *Nothobranchius furzeri* (killifish) | Xradia versa 620 (Zeiss, Dublin, CA)  HA phantom | 30 µm voxel size, WB: 601 projections, 180°; resolution, energy 60 kV, 110 mA, and 6.5 W with an air filter; caudal: 3 mm resolution, 4x magnification; 1,601 projections, 80 kV, 125 mA, 10 W, low energy filter | GRZ-AD, female, 5-, 9-, and 14-week-old | WB and caudal vertebrae  (Dragonfly Pro software) | centrum, neural spine, neural canal, neural  arch (NA), haemal spine, haemal canal, and haemal arch (HA);  BV, TV, BV/TV fraction, bone surface (BS), average bone thickness (B.Th); average cortical thickness (Ct.Th); length of C1, radius of centrum, neural and haemal arch angles and areas |
| (Sakashita et al., 2019) | 32 species from 10 orders of Teleostei | Skyscan 1172 (Bruker) | 2-14µm voxel size; 50–80 kV | adults (precise age not reported, sex not reported | vertebral body, first haemal arch | Thickness of the sheet-like trabeculae; plate-like ridge and internal hollow spaces |

**Table References**

ABE, K., INO, H., NIWA, T., SEMMY, D., TAKAOCHI, A., NISHIMURA, T., MOGI, C., UENAKA, M., ISHII, M., TANAKA, K., OHKAWA, Y. & ISHITANI, T. 2024. Sex-dependent regulation of vertebrate somatic growth and aging by germ cells. *Sci Adv,* 10**,** eadi1621.

BUTYLINA, M., FÖGER-SAMWALD, U., GAMSJAEGER, S., WAHL-FIGLASH, K., KOTHMAYER, M., PASCHALIS, E. P., PUSCH, O. & PIETSCHMANN, P. 2022. <b><i>Nothobranchius furzeri,</i></b> the Turquoise Killifish: A Model of Age-Related Osteoporosis? *Gerontology*.

CHARLES, J. F., SURY, M., TSANG, K., URSO, K., HENKE, K., HUANG, Y., RUSSELL, R., DURYEA, J. & HARRIS, M. P. 2017. Utility of quantitative micro-computed tomographic analysis in zebrafish to define gene function during skeletogenesis. *Bone,* 101**,** 162-171.

CHO, S. H., LEE, S., PARK, J. I., LA YANG, Y., KIM, S. R., AHN, J., JEONG, H., JUNG, H. Y., GWAK, N., KIM, K. N. & KIM, Y. 2023. Age-associated spinal stenosis in the turquoise killifish. *iScience,* 26**,** 107877.

HINO, H., KONDO, S. & KURODA, J. 2024. In vivo imaging of bone collagen dynamics in zebrafish. *Bone Rep,* 20**,** 101748.

HUR, M., GISTELINCK, C. A., HUBER, P., LEE, J., THOMPSON, M. H., MONSTAD-RIOS, A. T., WATSON, C. J., MCMENAMIN, S. K., WILLAERT, A., PARICHY, D. M., COUCKE, P. & KWON, R. Y. 2017. MicroCT-based phenomics in the zebrafish skeleton reveals virtues of deep phenotyping in a distributed organ system. *Elife,* 6.

KAGUE, E., TURCI, F., NEWMAN, E., YANG, Y., BROWN, K. R., AGLAN, M. S., OTAIFY, G. A., TEMTAMY, S. A., RUIZ-PEREZ, V. L., CROSS, S., ROYALL, C. P., WITTEN, P. E. & HAMMOND, C. L. 2021. 3D assessment of intervertebral disc degeneration in zebrafish identifies changes in bone density that prime disc disease. *Bone Research,* 9**,** 39.

KALIYA-PERUMAL, A. K., CELIK, C., CARNEY, T. J., HARRIS, M. P. & INGHAM, P. W. 2024. Genetic regulation of injury-induced heterotopic ossification in adult zebrafish. *Dis Model Mech,* 17.

MARCÉ-NOGUÉ, J. & LIU, J. 2020. Evaluating fidelity of CT based 3D models for Zebrafish conductive hearing system. *Micron,* 135**,** 102874.

MONMA, Y., SHIMADA, Y., NAKAYAMA, H., ZANG, L., NISHIMURA, N. & TANAKA, T. 2019. Aging-associated microstructural deterioration of vertebra in zebrafish. *Bone Rep,* 11**,** 100215.

OFER, L., DEAN, M. N., ZASLANSKY, P., KULT, S., SHWARTZ, Y., ZARETSKY, J., GRIESS-FISHHEIMER, S., MONSONEGO-ORNAN, E., ZELZER, E. & SHAHAR, R. 2019. A novel nonosteocytic regulatory mechanism of bone modeling. *PLOS Biology,* 17**,** e3000140.

RAMAN, R., ANTONY, M., NIVELLE, R., LAVERGNE, A., ZAPPIA, J., GUERRERO-LIMON, G., CAETANO DA SILVA, C., KUMARI, P., SOJAN, J. M., DEGUELDRE, C., BAHRI, M. A., OSTERTAG, A., COLLET, C., COHEN-SOLAL, M., PLENEVAUX, A., HENROTIN, Y., RENN, J. & MULLER, M. 2024. The Osteoblast Transcriptome in Developing Zebrafish Reveals Key Roles for Extracellular Matrix Proteins Col10a1a and Fbln1 in Skeletal Development and Homeostasis. *Biomolecules,* 14.

RAMLI, ARAMAKI, T., WATANABE, M. & KONDO, S. 2023. Piezo1 mutant zebrafish as a model of idiopathic scoliosis. *Front Genet,* 14**,** 1321379.

SAKASHITA, M., SATO, M. & KONDO, S. 2019. Comparative morphological examination of vertebral bodies of teleost fish using high-resolution micro-CT scans. *Journal of Morphology,* 280**,** 778-795.

SICCARDI, A. J., 3RD, PADGETT-VASQUEZ, S., GARRIS, H. W., NAGY, T. R., D'ABRAMO, L. R. & WATTS, S. A. 2010. Dietary strontium increases bone mineral density in intact zebrafish (Danio rerio): a potential model system for bone research. *Zebrafish,* 7**,** 267-73.

SUNIAGA, S., ROLVIEN, T., VOM SCHEIDT, A., FIEDLER, I. A. K., BALE, H. A., HUYSSEUNE, A., WITTEN, P. E., AMLING, M. & BUSSE, B. 2018. Increased mechanical loading through controlled swimming exercise induces bone formation and mineralization in adult zebrafish. *Scientific Reports,* 8**,** 3646.

WALDMANN, L., LEYHR, J., ZHANG, H., ALLALOU, A., OHMAN-MAGI, C. & HAITINA, T. 2021. The role of Gdf5 in the development of the zebrafish fin endoskeleton. *Dev Dyn*.

WATSON, C., MONTES DE OCA, E., FIEDLER, I., ROJAS, M., TANG, W., GARDINER, E., BUSSE, B., HSU, Y. & KWON, R. 2021. wnt16 exerts pleiotropic effects on bone and lean mass in zebrafish. *bioRvix*.
